# 手性多孔有机笼键合硅胶高效液相色谱固定相的制备及其用于拆分手性化合物

**DOI:** 10.3724/SP.J.1123.2025.06014

**Published:** 2025-12-08

**Authors:** Jialei WU, Liqin YU, Bangjin WANG, Shengming XIE, Junhui ZHANG, Liming YUAN

**Affiliations:** 云南师范大学化学化工学院，云南省现代分离分析与物质转化重点实验室，云南 昆明 650500; Yunnan Key Laboratory of Modern Separation Analysis and Substance Transformation，College of Chemistry and Chemical Engineering，Yunnan Normal University，Kunming 650500，China

**Keywords:** 高效液相色谱, 手性分离, 手性固定相, 多孔有机笼, high performance liquid chromatography （HPLC）, chiral separation, chiral stationary phase （CSP）, porous organic cage （POC）

## Abstract

多孔有机笼（POCs）是由分立的三维笼状分子通过分子间作用力组装而形成的一类新兴的多孔材料。它们具有良好的溶解性和明确的分子固有内腔，是一种良好的色谱分离介质。本研究以1，3，5-均苯三甲醛和（1*R*，2*R*）-环己二胺为原料合成了一种手性POC（CC3-R，C_72_H_84_N_12_），用NaBH_4_还原制得RCC3-R（C_72_H_108_N_12_）。将RCC3-R后修饰引入碳碳双键功能化的连接臂后通过巯基-烯点击反应将其键合到巯基硅胶表面，成功制备了一种高效液相色谱手性固定相（CSP）。在正相（NP）和反相（RP）两种洗脱模式下对手性醇类、酯类、酮类、醛类、胺类、有机酸类等不同类型的外消旋体进行了分离测试，评价了该CSP的手性识别能力。结果表明，该CSP在NP和RP洗脱模式下均表现出较好的手性拆分能力，分别实现了对18种和16种外消旋体的拆分。此外，该CSP填充柱还与两种广泛使用的商品柱（Chiralcel OD-H柱和Chiralpak AD-H柱）具有良好的手性拆分互补性，能够拆分这两种商品柱无法拆分的一些外消旋体。同时，还考察了柱温和进样量对自制的RCC3-R柱手性拆分性能的影响。该自制柱还具有较好的重复性和稳定性，经数百次进样使用后，对外消旋体拆分的保留时间和分离度与柱使用之初无显著变化，相对标准偏差（RSD，*n*=5）分别≤0.50%和≤1.30%，此外，不同批次制备的RCC3-R手性柱对外消旋体拆分的保留时间和分离度的RSD（*n*=3）分别≤1.89%和≤4.10%。本研究表明手性POC RCC3-R是一种具有潜在应用价值的高效液相色谱手性分离材料，也对基于手性POCs的新型高效液相色谱CSP的研究具有重要的意义。

## Abstract (en)

Porous organic cages （POCs） are a new class of porous materials formed by the assembly of discrete three-dimensional cage-like molecules through intermolecular forces. They possess good solubility and well-defined intrinsic molecular cavities， making them an extremely attractive medium for chromatographic separation. In this study， a chiral porous organic cage （CC3-R， C_72_H_84_N_12_） was synthesized using 1，3，5-triformylbenzene and （1*R*，2*R*）-diaminocyclohexane as raw materials， and it was then reduced by NaBH_4_ to obtain RCC3-R （C_72_H_108_N_12_）. After post-modification of RCC3-R with 5-bromo-1-pentene to introduce carbon-carbon double-bond functionalized linker arms， it was successfully bonded to the surface of thiolated silica via thiol-ene click reaction to prepare a chiral stationary phase （CSP） for high performance liquid chromatography （HPLC）. The successful synthesis of CC3-R， RCC3-R and CSP was confirmed by various characterization methods such as nuclear magnetic resonance （NMR）， Fourier-transform infrared spectroscopy （FT-IR）， high resolution mass spectrometry （HRMS）， thermogravimetric analysis （TGA）， and elemental analysis （EA）. The enantioseparation performance of the CSP was evaluated by separating various types of racemic compounds， including alcohols， esters， ketones， aldehydes， amines， and organic acids in both normal-phase （NP） and reversed-phase （RP） elution modes. The results demonstrate that the CSP achieved enantioseparation of 18 and 16 racemates in NP and RP modes， respectively. Among them， 12 racemates achieved baseline separation in NP elution mode， while 7 racemates achieved baseline separation in RP elution mode. Moreover， the CSP-packed column exhibited good complementarity in chiral separation with two widely used commercial columns， Chiralcel OD-H and Chiralpak AD-H， enabling the separation of some racemic compounds that cannot be separated on these two commercial columns. In NP elution mode， 7 of the 18 tested racemates could not be enantioseparated on Chiralpak AD-H column， and 4 of the 18 tested racemates could not be enantioseparated on Chiralcel OD-H column. In RP elution mode， 8 of the 16 tested racemates could not be enantioseparated on Chiralpak AD-H column， and 5 of the 16 tested racemates could not be enantioseparated on Chiralcel OD-H column. The effects of column temperature and injection mass on chiral separation performance of the RCC3-R column were investigated. In addition， the RCC3-R column exhibited excellent repeatability and stability. After hundreds of injections， no significant changes were observed in the retention times and resolution values of the analytes compared to the initial use of the column， with relative standard deviations （RSDs， *n*=5） of less than 0.50% and 1.30%， respectively. Furthermore， the RSDs （*n*=3） of retention times and resolution values of the racemates separated on RCC3-R columns prepared in different batches were less than 1.89% and 4.10%， respectively. This study demonstrates that the chiral POC RCC3-R is a potentially valuable chiral separation material for HPLC. It also holds significant importance for the research on novel CSPs for HPLC based on chiral POCs.

由于手性化合物的不同构型对映体在生物体内的生物活性、代谢过程和毒性存在显著差异，因此获取单一构型的对映体是有机合成、医药生产、农业、食品等领域的研究热点^［1，2］^。目前，基于手性固定相（CSPs）的高效液相色谱法（HPLC）被认为是制备、分析和拆分外消旋体最有效和最广泛使用的方法之一^［3，4］^。CSPs的性能直接决定着手性分离的选择性和效率，因此开发具有优秀手性识别能力的新型CSPs对于手性分离领域来说具有重要的意义。近年来，多孔材料如金属有机框架（MOFs）^［5-7］^、共价有机框架（COFs）^［8，9］^和微孔有机网状聚合物（MONs）^［10］^因其具有较高的表面积和明确的多孔结构，已经被作为CSPs广泛用于HPLC手性分离。

多孔有机笼（POCs）是由分立的三维笼状分子通过分子间作用力组装而成的一类新型多孔材料^［11，12］^，凭借独特的笼状分子结构、明确的分子内腔多孔性等特点，其在分子识别、分离、催化、传感、气体吸附等领域展现出巨大的应用潜力^［13-15］^。与其他多孔框架材料（如MOFs、COFs和MONs）相比，POCs是通过弱的分子间作用力而非强的配位键或共价键将分立的笼状分子组装形成多孔固体，因此POCs具有良好的溶解性和溶液加工性，使其易于进行结构后修饰、化学键合和溶液涂敷^［16］^，是一类良好的色谱分离材料。2015年，本课题组将一种手性POC（CC3-R）作为CSP涂覆于毛细管内壁制备了气相色谱手性柱^［17］^，该手性柱展现出优异的对映体分离性能，数十种外消旋体在该柱上获得了拆分，证实了手性POCs是一种具有良好应用前景的气相色谱CSP。至此之后，许多手性POCs被用作CSP涂覆在毛细管内壁上制备气相色谱柱用于对映体分离，如CC9^［18］^、CC10^［19］^、NC1-R^［20］^、戊基手性POC^［21］^、羟基手性POC^［22，23］^、柱状手性POC^［24，25］^等。这些手性POCs在气相色谱中展现出优秀的手性分离效果，并且它们之间还存在良好的手性拆分互补性，还能拆分商品*β*-DEX 120气相色谱手性柱不能拆分的许多外消旋体，展现良好的应用前景。POCs是由轻质元素（如C、H、N等）组成的纯有机笼状小分子通过分子间作用力自组装形成的多孔固体，其密度通常较小，并具有良好的溶解性。当它用作HPLC固定相时，需要将其键合在硅胶基质上制备CSP，制备过程相对复杂，并要求手性POCs具有可用于键合反应的活性官能团。然而，合成具有活性官能团（如-OH、-NH_2_、-COOH）的手性POCs仍然是一个挑战，目前报道的具有上述活性官能团的手性POCs较少。因此，将手性POCs用于HPLC手性分离相对滞后，并且研究较少。直到2022年，本课题组通过化学反应将一种具有羟基功能基团的手性POC（NC1-R）键合在硅胶基质表面，制备了HPLC固定相^［26］^，实现了多种外消旋体的有效拆分，证明了POCs作为HPLC手性分离介质的应用潜力。此后，一些手性POCs，如NC4-R^［27］^、CC19-R^［28］^、CC3-R^［29，30］^和RCC2-S^［31］^也被用于HPLC对映体分离，并展现出优秀的手性识别能力，但将手性POCs用于HPLC对映体分离仍然处于早期探索阶段。因此，进一步研究具有不同手性拓扑结构、空腔和手性识别位点的手性POCs用于HPLC对映体分离，从而拓展可分离手性化合物的种类，这对发展基于POCs的HPLC新型CSPs具有重要意义。

点击化学是Sharpless于2001年首次提出的一种绿色、高效的有机合成方法^［32］^，它能够将反应单元快速且可靠地连接起来，从而高效地完成目标分子的化学合成。巯基-烯点击反应作为点击化学中的重要分支，因其优异的反应选择性和温和的反应条件，已被广泛应用于色谱固定相的制备^［26，33，34］^。

本文以1，3，5-均三苯甲醛和（1*R*，2*R*）-环己二胺为原料，通过一步亚胺缩合反应制备了手性POC CC3-R，并还原其亚胺键得到RCC3-R。随后，对RCC3-R进行烯基功能化修饰后利用巯基-烯点击合成策略将其键合到巯基硅胶表面，制得了一种HPLC CSP。在正相（NP）和反相（RP）洗脱模式下，通过对多种类型的外消旋体进行拆分测试，评价了该CSP填充色谱柱的手性分离性能，并与两种市售商品柱Chiralcel OD-H和Chiralpak AD-H进行了比较。此外，还考察了柱温和进样量对手性拆分效果的影响，并评价了色谱柱的重复性和稳定性。

## 1 实验部分

### 1.1 仪器、试剂和材料

LC-16高效液相色谱仪（日本Shimadzu公司）；1666型高效液相色谱装柱机（美国Alltech公司）；Avance NEO 500 MHz核磁波谱仪（NMR，德国Bruker公司）；Nicolet iS20光谱仪（FT-IR）、LTO-Orbitrap XL高分辨质谱仪（HRMS，美国Thermo Fisher公司）；SDT-650热分析仪（TGA，美国Taber公司）；Vario EL Ⅲ元素分析仪（EA，德国Elementar公司）。

1，3，5-均苯三甲醛（纯度99%）、（1*R*，2*R*）-环己二胺（纯度99%）、5-溴-1-戊烯（纯度99%）、偶氮二异丁腈（AIBN，纯度98%）、三氟乙酸（TFA，纯度99%）、（3-巯基丙基）三甲氧基硅烷（纯度98%）和氢化钠（NaH，纯度60%）购自中国上海Adamas-beta试剂公司；硼氢化钠（NaBH_4_，纯度98%）购自中国天津市福晨化学试剂厂；无水硫酸镁（MgSO_4_，纯度99.5%）购自中国上海Aladdin试剂公司；二氯甲烷（DCM，纯度99.5%）、乙醇（EtOH，纯度99.7%）、三氯甲烷（CHCl_3_，纯度99%）、甲醇（MeOH，纯度99.5%）、甲苯（纯度99.5%）、吡啶（纯度99.5%）和丙酮（纯度99.5%）购自中国天津风船化学试剂科技有限公司；甲醇（色谱纯）、正己烷（*n*-HEX，色谱纯）、异丙醇（IPA，色谱纯）购自中国上海Sigma-Aldrich试剂公司；测试的外消旋体（纯度≥98%）购自中国上海Adamas-beta、TCI和Aladdin试剂公司。

球形硅胶（5 µm， 12 nm， 300 m^2^/g）购自中国苏州纳微生物科技有限公司；Chiralpak AD-H色谱柱（250 mm×4.6 mm）和Chiralcel OD-H色谱柱（250 mm×4.6 mm）购自中国上海大赛璐药物手性技术有限公司；HPLC空管（250 mm×2.1 mm）购自中国大连日普利科技仪器有限公司。

### 1.2 实验过程

#### 1.2.1 CC3-R和RCC3-R的合成

CC3-R^［11］^和RCC3-R^［35］^的合成路线如图1所示。在室温条件下，将1，3，5-均苯三甲醛固体（5.0 g，30.86 mmol）缓慢加入到100 mL DCM中，此过程无需搅拌。随后，向反应体系中加入100 μL TFA作为亚胺缩合反应的催化剂。接着，缓慢加入溶有（1*R*，2*R*）-环己二胺（5.0 g，44.64 mmol）的100 mL DCM溶液。反应体系在室温下静置5天，此间反应容器两侧逐渐有晶体析出。通过过滤收集结晶产物，并用EtOH-DCM（95∶5，体积比）溶液多次洗涤，最终获得CC3-R晶体。

**图1:**
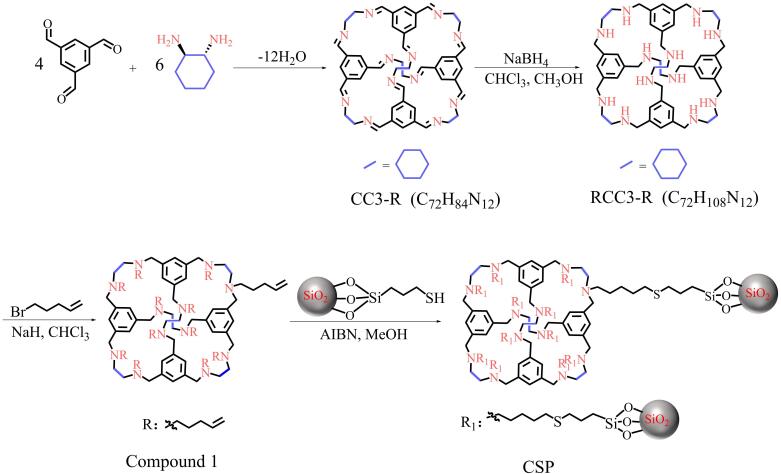
CC3-R、RCC3-R和CSP的合成

将合成的CC3-R（0.92 g，0.81 mmol）溶解在50 mL CHCl_3_-MeOH（50∶50，体积比）中，搅拌至溶液完全澄清。在室温条件下，向上述溶液中缓慢加入NaBH_4_（0.98 g，27 mmol），并持续搅拌12 h。随后，向反应体系中加入3 mL水猝灭反应，继续搅拌12 h。反应结束后，除去反应溶剂，得到固体产物。将固体产物溶解在50 mL CHCl_3_中，用去离子水洗涤（3×50 mL），收集有机相，并用无水MgSO_4_干燥。过滤除去MgSO_4_后，通过旋转蒸发除去溶剂得到白色的还原固体产物RCC3-R。

#### 1.2.2 CSP的制备

利用巯基-烯点击反应制备CSP，合成示意图见图1。首先，根据文献报道的方法制备巯基硅胶（SiO_2_-SH）^［36］^。将球形硅胶（5.0 g）分散至100 mL 10%的HCl溶液中，100 ℃真空条件下回流24 h，抽滤，得到的固体用去离子水反复洗涤至洗涤液呈中性。随后将固体80 ℃真空干燥过夜得到活化硅胶（SiO_2_）。氮气（N_2_）氛围下，将活化硅胶（4.0 g）和无水甲苯（70 mL）置于250 mL圆底烧瓶中，搅拌至硅胶分散均匀后，向体系中依次加入4 mL （3-巯基丙基）三甲氧基硅烷和2 mL无水吡啶。随后将反应温度缓慢升至100 ℃，并在此温度下回流72 h。反应结束并冷却至室温后，抽滤，得到的固体依次用甲苯、丙酮、甲醇洗涤，最后真空干燥8 h得到SiO_2_-SH。

在N_2_氛围下，将NaH（0.50 g，25.00 mmol）和无水CHCl_3_（50 mL）置于100 mL圆底烧瓶中并在冰浴条件下持续搅拌20 min。接着逐滴加入10 mL溶有RCC3-R（0.50 g，0.72 mmol）的无水CHCl_3_溶液，继续在冰浴条件下搅拌30 min。随后向体系中加入0.8 mL 5-溴-1-戊烯并缓慢升温至60 ℃，在此温度下回流72 h。反应结束后，过滤，滤液加入30 mL去离子水，用CHCl_3_（3×50 mL）萃取，有机相用无水MgSO_4_干燥，抽滤后的溶液通过旋蒸得到碳碳双键功能化的RCC3-R（化合物1）。

在N_2_氛围下，将化合物1（0.50 g）、SiO_2_-SH（1.50 g）、AIBN（0.07 g）和MeOH（50 mL）加入100 mL圆底烧瓶中，缓慢升温至60 ℃回流72 h。反应结束并冷却至室温后，除去溶剂，得到的固体依次用MeOH、DCM和丙酮依次洗涤。最后将固体产物在70 ℃真空干燥8 h，得到基于RCC3-R的CSP。

#### 1.2.3 色谱柱的填充和HPLC条件

利用超声处理，将CSP（1.3 g）均匀分散至30 mL *n*-HEX-IPA（90∶10，体积比）中，得到匀浆液。在50 MPa高压下，以*n*-HEX-IPA（90∶10，体积比）为推进溶剂将匀浆液填入HPLC空管。5 min后，将系统压力调至25 MPa，保持30 min。填充结束后，将填充柱接入HPLC系统，用*n*-HEX-IPA（90∶10，体积比）作为流动相，以0.1 mL/min的流速洗脱至基线平稳即可进行HPLC测试。测试条件：正相洗脱模式以不同体积比的*n*-HEX-IPA为流动相，反相洗脱模式以不同体积比的MeOH-H_2_O为流动相；流速：0.1 mL/min；测试波长：254 nm；柱温：25 ℃。在NP-HPLC模式下，用苯测得其理论板数为14 600 块/m；在RP-HPLC模式下，用萘测得其理论板数为13 200 块/m。

## 2 结果与讨论

### 2.1 CC3-R、RCC3-R和CSP的表征

采用HRMS、NMR、FT-IR、TGA、EA等方法对CC3-R、RCC3-R和CSP进行了表征。CC3-R的分子式为C_72_H_84_N_12_，其理论相对分子质量（*M*_r_）为1 117.517 8。在CC3-R的HRMS图中（图2a），*m*/*z*=1 118.707 8和*m*/*z*=559.358 1处的信号峰分别为CC3-R的［M+H］⁺和［M+2H］²⁺离子峰。对CC3-R进行了NMR表征，核磁数据如下，^1^H NMR（500 MHz，CDCl_3_）：*δ* 8.19（s，CH=N，12H），7.93（s，ArH，12H），3.34~3.41（m，CH on cyclohexane，12H），1.48~1.88（m，CH_2_ on cyclohexane，48H）；^13^C NMR（125 MHz，CDCl_3_）：*δ* 159.18，136.61，129.60，74.65，33.04，24.41（谱图见附图S1，https://www.chrom-China.com）。通过NaBH_4_将CC3-R的亚胺键（C=N）还原得到RCC3-R，其分子式为C_72_H_108_N_12_，计算得到其理论*M*_r_为1 141.882 0。在RCC3-R的HRMS图中（图2b），*m*/*z*=571.453 6和*m*/*z*=381.303 0处的信号峰分别为RCC3-R的［M+2H］^2^⁺和［M+3H］^3^⁺的离子峰。RCC3-R的NMR数据如下，^1^H NMR（500 MHz，CDCl_3_）：*δ* 7.19（s，ArH，12H），3.89（d，ArCH_2_，12H），3.64（d，ArCH_2_，12H，），2.24~2.29（m，NH，12H），2.05~2.08（m，CH on cyclohexane，12H），1.03~1.73（m，CH_2_ on cyclohexane，48H）；^13^C NMR（125 MHz，CDCl_3_）：*δ* 141.40，124.98，61.39，50.74，31.81，25.03（谱图见附图S2）。以上表征结果证明CC3-R和RCC3-R的成功合成。

**图2:**
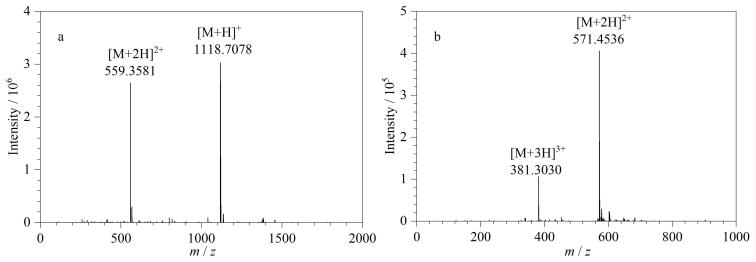
（a）CC3-R和（b）RCC3-R的HRMS图

CC3-R、RCC3-R、SiO_2_-SH和CSP的FT-IR图如图3a所示。与SiO_2_-SH相比，CSP在2 930 cm^-1^和2 850 cm^-1^处的吸收峰强度显著增强，这是由SiO_2_-SH表面键合的RCC3-R中大量饱和的-CH_2_-和-CH-基团中的C-H伸缩振动引起的。此外，CSP在1 600 cm^-1^和1 450 cm^-1^处出现了新的吸收峰，这归因于RCC3-R分子结构中苯环的骨架振动。从SiO_2_、SiO_2_-SH和CSP的TGA图（图3b）可以看出，与SiO_2_-SH相比，CSP的质量损失更为显著，这主要是由于硅胶表面键合的RCC3-R有机成分的分解所致。这一结果进一步证实通过巯基-烯点击反应成功将RCC3-R键合到了SiO_2_-SH表面。此外，元素分析数据（表1）显示，与SiO_2_-SH相比，CSP的C、N和H含量显著增加，这也证实了RCC3-R与SiO_2_-SH的成功键合。根据表面键合量计算公式^［37］^计算得到CSP的表面键合量为0.22 μmol/m^2^。

**图3:**
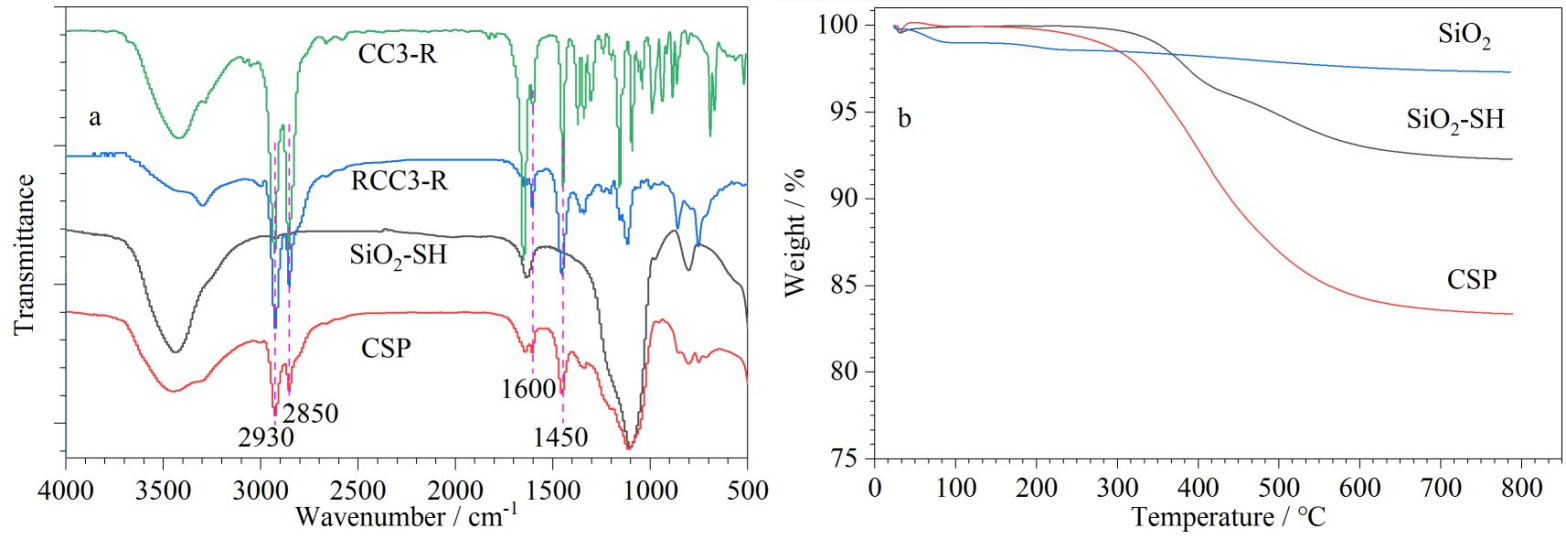
（a）CC3-R、RCC3-R、SiO_2_-SH和CSP的FT-IR图；（b）SiO_2_、SiO_2_-SH和CSP的TGA图

**表1:** SiO_2_-SH和CSP的元素分析数据

Sample	Contents/%		
	C	N	H
SiO_2_-SH	4.36	˂0.05	1.79
CSP	10.02	1.12	2.04

### 2.2 正相洗脱模式下外消旋体的拆分

为评估该CSP在NP洗脱模式下的对映体分离性能，选取了多种类型的外消旋化合物（包括醇、酮、酯、醛、酸、胺类等）作为分离对象，采用不同比例的正己烷/异丙醇作为流动相进行对映体分离实验。结果表明，在NP洗脱模式下，该CSP填充色谱柱共对18种外消旋体（分子结构见附图S3）展现出良好的手性拆分能力，相应的色谱评价参数详见表2，分离色谱图见图4。由表2可知，被拆分的这些外消旋体中，共有12种达到基线分离（*R*_s_>1.5），表明该色谱柱具有良好的手性识别能力。其中1-苯基乙胺外消旋体（*R*_s_=5.86）、4，4′-二甲基安息香外消旋体（*R*_s_=5.19）、3-苄氧基-1，2-丙二醇外消旋体（*R*_s_=4.66）和1，1′-联-2-萘酚外消旋体（*R*_s_=4.09）等获得了较高的*R*_s_值。此外，还将这18种外消旋体在两种常用商品柱（Chiralpak AD-H和Chiralcel OD-H）上进行了拆分，并对3种色谱柱的拆分效果进行了对比分析，对比数据列于表2，对比色谱图列于附图S4。数据显示，在这18种外消旋体中，分别有7种和4种分析物未能在Chiralpak AD-H柱和Chiralcel OD-H柱上实现拆分。其中，这两种商品柱均无法拆分3-羟基-2-丁酮、1-（3-氟苯基）乙醇和布洛芬这3种外消旋体，但该制备的RCC3-R手性柱能较好地拆分这3种外消旋体。这一结果进一步凸显了该色谱柱在手性分离中的潜力。同时，一些外消旋体在这两种商品柱上的拆分效果也不如RCC3-R手性柱，如4，4′-二甲基安息香、3-苄氧基-1，2-丙二醇、1，1′-联-2-萘酚、4，4′-二氟安息香和1-（4-氯苯基）乙醇这5种外消旋体在RCC3-R手性柱上拆分的*R*_s_值均明显高于它们在两个商品柱上拆分的*R*_s_值。这些结果表明，该色谱柱与两种商品柱间具有良好的手性拆分互补性，可以拆分两种商品柱无法拆分或者拆分效果不好的部分外消旋体。

**表2:** NP洗脱模式下外消旋体在RCC3-R、Chiralpak AD-H和Chiralcel OD-H柱上的分离数据

Racemate	RCC3-R			Chiralpak AD-H			Chiralcel OD-H		
	*k*_1_	*α*	*R*_s_	*k*_1_	*α*	*R*_s_	*k*_1_	*α*	*R*_s_
1-Phenylethylamine	0.90	2.54	5.86^a）^	0.71	1.00	-	0.96	1.19	0.81
4，4′-Dimethylbenzoin	0.81	2.58	5.19^a）^	5.06	1.10	2.74	1.69	1.36	2.39
3-（Benzyloxy）propane-1，2-diol	3.51	1.86	4.66^a）^	2.24	1.19	0.70	2.73	1.06	2.12
1，1′-Bi-2-naphthol	3.39	1.56	4.09^b）^	7.44	1.03	0.10	3.80	1.11	1.25
4，4′-Difluorobenzoin	1.37	2.44	4.00^a）^	4.36	1.10	2.09	1.81	1.09	0.93
3-Hydroxy-2-butanone	1.57	1.52	3.06^a）^	1.29	1.00	-	0.84	1.00	-
1-（4-Chlorophenyl）ethanol	2.61	1.28	2.29^b）^	0.86	1.04	0.49	0.78	1.13	0.65
Methyl mandelate	0.77	1.62	1.93^a）^	1.92	1.08	1.63	1.45	2.04	7.68
1-（3-Bromophenyl）ethanol	1.03	1.45	1.91^a）^	1.04	1.00	-	0.86	1.13	0.71
1-（3-Fluorophenyl）ethanol	0.90	1.40	1.69^a）^	0.83	1.00	-	0.62	1.00	-
3-Chloro-2-butanone	1.17	1.30	1.62^a）^	0.40	2.31	0.83	0.33	1.97	3.02
2-Methoxy-2-phenylethanol	0.89	1.31	1.53^a）^	0.91	1.23	2.70	0.81	1.13	0.69
2，2′-Furoin	1.35	1.23	1.30^b）^	2.08	1.00	-	3.43	1.26	3.54
Dihydroflavone	1.17	1.23	1.28^a）^	1.45	1.07	1.16	1.55	1.42	3.66
Ibuprofen	0.62	1.42	1.27^a）^	0.71	1.00	-	0.69	1.00	-
2-Ethylhexanoic acid	1.08	1.30	1.26^a）^	0.40	1.41	1.13	0.66	1.00	-
Ethyl mandelate	1.65	1.23	0.94^a）^	1.92	1.08	1.63	1.45	2.04	7.68
1-Phenylethanol	0.91	1.18	0.87^a）^	0.84	1.00	-	0.90	1.22	1.29

The mobile phases employed for resolving each racemate on the RCC3-R column is annotated with superscripts a） or b） to the right of the corresponding *R*_s_ values， where a） denotes *n*-hexane （*n*-HEX）-isopropanol （IPA） （90∶10， volume raio） and b） denotes *n*-HEX-IPA （95∶5， volume raio）. All the racemates separated on the Chiralpak AD-H and Chiralcel OD-H columns using *n*-HEX-IPA （90∶10， volume raio） as mobile phase. Flow rate： 0.1 mL/min for the RCC3-R column， and 0.5 mL/min for Chiralpak AD-H and Chiralcel OD-H columns. *k*_1_：retention factor of the first enantiomer； *α*： separation factor； *R*_s_： resolution value of a pair of enantiomers； -： could not be separated.

**图4:**
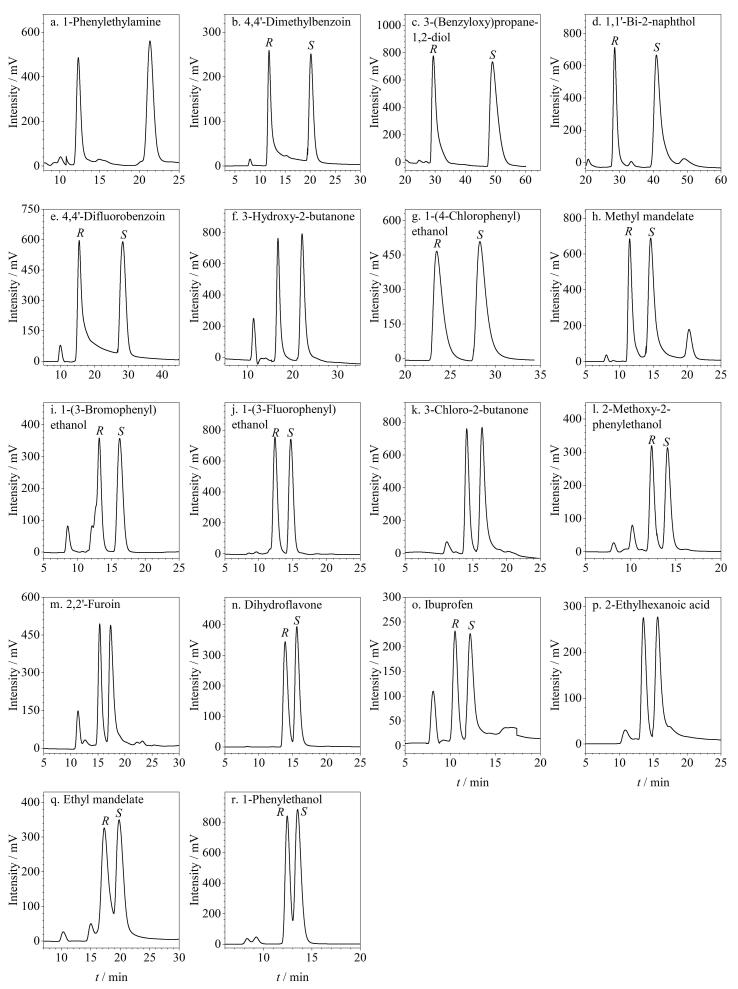
NP洗脱模式下外消旋体在RCC3-R柱上的色谱图

该CSP展现出显著的手性分离能力，这与RCC3-R的分子结构密不可分。合成的CC3-R是一种具有三重对称轴的四面体笼状分子（图5a），其每个面具有孔径约为0.92 nm的类似于三角形的孔窗（图5b），为分子内部与外部的物质交换提供了通道。更关键的是，CC3-R分子笼以对映体（1*R*，2*R*）-环己二胺作为手性构建模块，从而赋予了每个CC3-R分子固有的手性。每个分子的中心都具有一个内部空腔（图5a），分析物可以通过孔窗进入其中，并在分子笼内部发生丰富的主-客体相互作用，这种相互作用对于实现对映体分离至关重要。RCC3-R分子中含有大量的氢键作用位点（N原子），而大多数被分离的外消旋体中都含有氢键供体基团，如羟基（-OH）、羧基（-COOH）和氨基（-NH_2_）。这些基团能够与RCC3-R的N原子形成强氢键相互作用，从而显著增强了手性分离的效果。例如，3-羟基-2-丁酮和3-氯-2-丁酮的结构极为相似，唯一的区别在于前者的取代基为-OH，而后者的取代基为-Cl，-OH与RCC3-R之间的氢键作用强于-Cl。因此，3-羟基-2-丁酮与CSP之间的氢键作用更强，从而获得了更高的分离度（*R*_s_=3.06），而3-氯-2-丁酮的分离度则相对较低（*R*_s_=1.62）。此外，RCC3-R还含有大量的苯环，而许多分析物分子中也存在共轭结构，这些结构能够与CSP形成*π-π*相互作用。例如，4，4′-二甲基安息香和2，2′-联糠醛的结构相似，但前者含有两个苯环，而后者含有两个呋喃环，由于苯环的*π-π*相互作用强于呋喃环，因此4，4′-二甲基安息香在该CSP上获得了较高的分离度（*R*_s_=5.19），而2，2′-联糠醛（*R*_s_=1.30）则无法实现基线分离，这表明，分析物与CSP之间的*π-π*相互作用在手性分离过程中也发挥着重要作用。实际上，CSP与对映体之间的手性分离机制是一个复杂的过程，涉及手性微环境和多种主客体相互作用，难以进行精确阐述。总体而言，外消旋体在CSP上的分离主要得益于对映体与RCC3-R之间的氢键和*π-π*相互作用。此外，偶极-偶极相互作用、静电相互作用以及范德华力等也可能对手性拆分起到积极的促进作用。

**图5:**
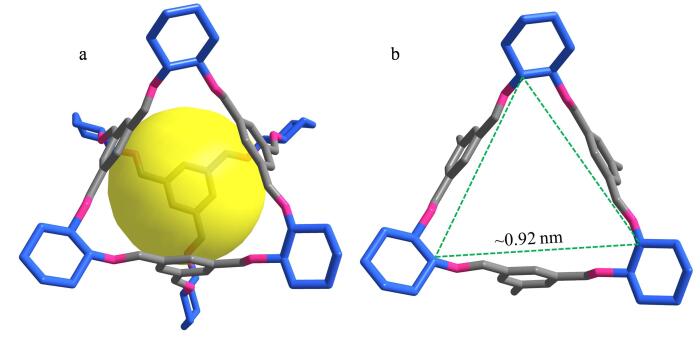
（a）CC3-R分子结构示意图及其（b）一个孔窗口的示意图

### 2.3 反相洗脱模式下外消旋体的拆分

为了进一步探究该CSP的性能，以MeOH-H_2_O作为流动相，对其在RP洗脱模式下的手性拆分能力进行了考察。实验结果表明，在这种洗脱模式下，该CSP成功实现了16种外消旋体（分子结构见附图S5）的拆分，这些化合物涵盖了醇、酮、酯、醛、羧酸等多种类型。详细的实验数据和色谱图分别列于表3和图6中。在这些被分离的化合物中，有7种实现了基线分离，其中拆分反-1，2-二苯基环氧乙烷外消旋体（*R*_s_=6.51）、布洛芬外消旋体（*R*_s_=2.88）等的*R*_s_值较高，表明该CSP在RP-HPLC中也具有较好的手性识别能力。在被该CSP拆分的17种外消旋体中，有8种在Chiralpak AD-H色谱柱上无法实现拆分，5种在Chiralcel OD-H色谱柱上无法实现拆分（表3），并且部分外消旋体在RCC3-R手性柱上获得了比在两种商品柱上更高的*R*_s_值，显示出更好的对映体分离效果。例如，扁桃酸乙酯外消旋体在RCC3-R手性柱上拆分的*R*_s_值为1.84，而在Chiralpak AD-H色谱柱上拆分的*R*_s_值为1.63；反-1，2-二苯基环氧乙烷外消旋体、1-（3-溴苯基）乙醇外消旋体、4，4′-二氟安息香外消旋体和2-甲氧基-2-苯乙醇外消旋体在RCC3-R手性柱上拆分的*R*_s_值分别为6.51、1.45、1.33和0.98，而这4种外消旋体在Chiralcel OD-H色谱柱上拆分的*R*_s_值分别为4.38、0.71、0.93和0.69。它们的对比色谱图列于附图S6。综上所述，该基于RCC3-R的CSP在NP和RP洗脱模式下均展现出较好的对映体分离性能，显示出RCC3-R在HPLC中的潜在应用价值。

**表3:** RP洗脱模式下外消旋体在RCC3-R、Chiralpak AD-H和Chiralcel OD-H柱上的分离数据

Racemate	RCC3-R			Chiralpak AD-H			Chiralcel OD-H		
	*k*_1_	*α*	*R*_s_	*k*_1_	*α*	*R*_s_	*k*_1_	*α*	*R*_s_
*trans*-Stilbene oxide	0.36	5.96	6.51^a）^	0.66	2.78	9.54	0.83	1.95	4.38
Ibuprofen	0.37	2.87	2.88^a）^	0.71	1.00	-	0.69	1.00	-
Propylene oxide	0.42	2.32	2.66^a）^	0.53	1.00	-	0.65	1.00	-
2，2，2-Trifluoro-1-（9-anthryl）ethanol	3.60	1.52	2.47^b）^	1.76	1.52	3.30	1.97	3.10	11.10
Ethyl mandelate	0.51	1.97	1.84^b）^	1.92	1.08	1.63	1.45	2.04	7.68
Omeprazole	1.17	1.45	1.52^b）^	4.53	1.26	2.53	6.84	1.30	2.77
1-（3-Bromophenyl）ethanol	0.67	1.64	1.53^a）^	1.04	1.00	-	0.86	1.13	0.71
*β*-Butyrolactone	0.36	1.71	1.37^a）^	0.57	1.00	-	0.65	1.00	-
4，4′-Difluorobenzoin	0.32	2.07	1.33^a）^	4.36	1.10	2.09	1.81	1.09	0.93
1，2-Diphenyl-1，2-ethanediol	0.63	1.48	1.29^b）^	0.36	1.00	-	2.61	1.00	-
4，4′-Dimethoxybenzoin	0.81	1.55	1.19^b）^	4.84	1.71	4.91	5.67	1.12	1.86
2-Methoxy-2-phenylethanol	0.46	1.54	0.98^b）^	0.91	1.23	2.70	0.81	1.13	0.69
2-Phenylcyclohexanone	0.65	1.34	0.77^a）^	0.89	1.00	-	1.23	1.15	1.22
2-Methylpentanal	0.48	1.38	0.76^a）^	0.47	1.00	-	0.44	1.00	-
3-（Benzyloxy）propane-1，2-diol	0.46	1.64	0.63^b）^	2.24	1.19	0.70	2.73	1.06	2.12
1-Phenyl-1，2-ethanediol	0.51	1.31	0.52^a）^	2.18	1.00	-	1.72	1.11	0.93

The mobile phases employed for resolving each racemate on the RCC3-R column is annotated with superscripts a） or b） to the right of the corresponding *R*_s_ values， where a） denotes MeOH-H_2_O （80∶20， volume raio） and b） denotes MeOH-H_2_O （70∶30， volume raio）. All the racemates separated on the Chiralpak AD-H and Chiralcel OD-H columns using *n*-HEX-IPA （90∶10， volume raio） as mobile phase. Flow rate： 0.1 mL/min for the RCC3-R column， and 0.5 mL/min for Chiralpak AD-H and Chiralcel OD-H columns.

**图6:**
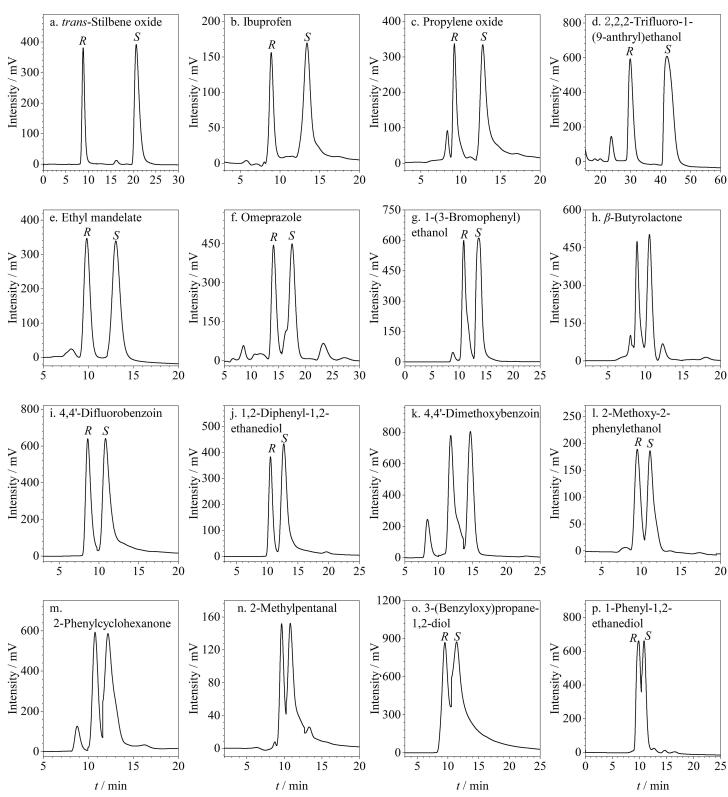
RP洗脱模式下外消旋体在RCC3-R柱上的色谱图

### 2.4 柱温对手性拆分的影响

为了研究温度对手性拆分性能的影响，在25~45 ℃不同柱温条件下对二氢黄酮外消旋体和1，2-二苯基-1，2-乙二醇外消旋体进行拆分测试（图7）。从获得的色谱图可以发现，随着柱温的升高，拆分这两种分析物对映体的保留时间逐渐减小，表明这些对映体在RCC3-R柱上的分离过程是一个放热过程。同时，对映体的van’t Hoff图显示出良好的线性关系（见附图S7），表明在研究温度范围内手性分离机制未发生改变。计算的热力学参数列于表4中。两对对映体的Δ*G*值均为负值，表明这些对映体从流动相转移到CSP的过程是一个热力学自发过程。Δ*G*的负值越大，对映体在CSP中保留时间越长，这与这两组外消旋体的洗脱顺序一致（两种分析物的洗脱顺序均为*R*-对映体早于*S*-对映体）。

**图7:**
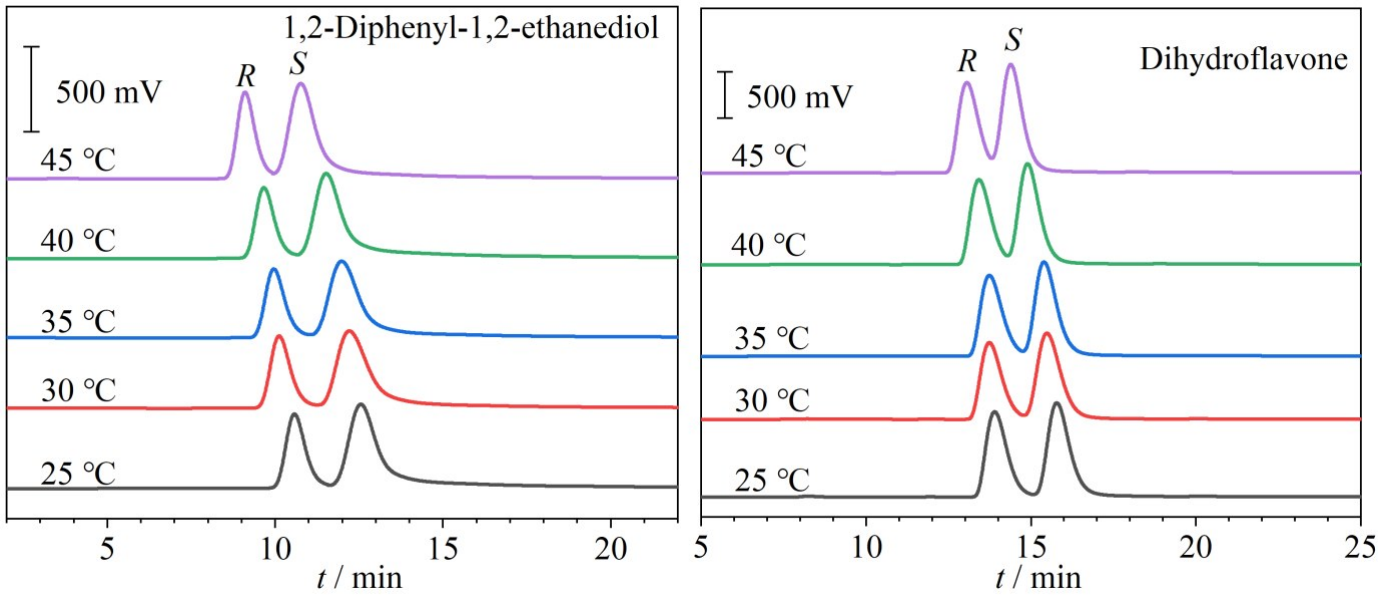
柱温对RCC3-R柱手性拆分效果的影响

**表4:** NP-HPLC下拆分二氢黄酮外消旋体和RP-HPLC下拆分1，2-二苯基-1，2-乙二醇外消旋体的热力学数值

Analyte	Δ*H*/ （kJ/mol）	Δ*S*/ （J/（mol·K）	Δ*G*/ （kJ/mol）
*R*-Dihydroflavone	-6.79±0.12	-10.25±0.40	-3.74±0.12
*S*-Dihydroflavone	-8.10±0.12	-12.98±0.38	-4.23±0.12
*R*-1，2-Diphenyl-1，2-ethanediol	-11.50±0.22	-31.94±0.71	-1.98±0.22
*S*-1，2-Diphenyl-1，2-ethanediol	-9.45±0.08	-19.27±0.23	-3.71±0.08

### 2.5 进样量对手性拆分的影响

为了探究分析物进样量对手性拆分效果的影响，在25 ℃柱温条件下，将二氢黄酮外消旋体和1，2-二苯基-1，2-乙二醇外消旋体的进样量从2 μg逐步增加到20 μg，得到的色谱图见图8。随着进样量的增加，拆分这两种分析物对映体的保留时间和分离效果均未发生显著变化（见附图S8）。此外，分析物单一构型对映体的峰面积随进样量的增加呈现出良好的线性增长关系（见附图S8）。在NP-HPLC条件下，该RCC3-R柱对二氢黄酮外消旋体的柱容量约为30 μg；在RP-HPLC条件下，该RCC3-R柱对1，2-二苯基-1，2-乙二醇外消旋体的柱容量约为35 μg。以上结果显示，该手性填充柱不仅在一定的进样量范围内能够保持较稳定的分离能力，还具有良好的柱容量和较好的定量分析性能。

**图8:**
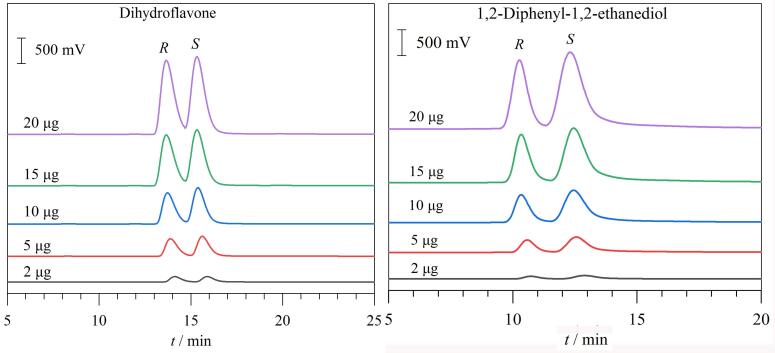
进样量对RCC3-R柱手性拆分效果的影响

### 2.6 柱的重复性和稳定性

手性柱的重复性和稳定性对对映体的分离具有重要意义。实验以二氢黄酮外消旋体和1，2-二苯基-1，2-乙二醇外消旋体为分析物研究了RCC3-R手性柱的重复性和稳定性。经过数百次进样使用后，拆分这两种分析物对映体的保留时间和分离度与柱使用之前几乎不变（图9），其相对标准偏差（RSD，*n*=5）分别≤0.50%和≤1.30%。此外，还考察了该手性色谱柱的柱间重复性。以二氢黄酮外消旋体和1，2-二苯基-1，2-乙二醇外消旋体为分析物，在NP和RP条件下在3根不同批次制备的固定相填充色谱柱（柱1、2和3）上进行了分离测试（色谱图见附图S9），拆分它们的对映体的保留时间和分离度的RSD（*n*=3）分别≤1.89%和≤4.10%。以上结果表明该色谱柱具有良好稳定性和重复性。

**图9:**
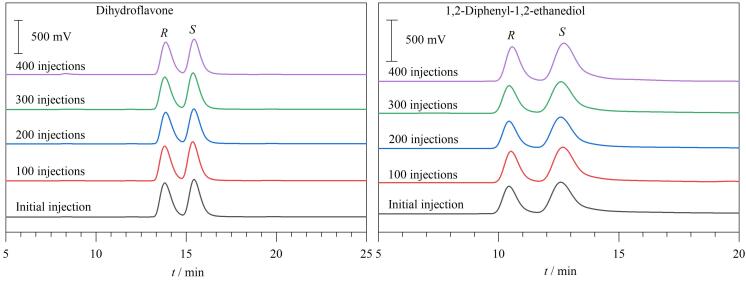
RCC3-R柱的进样重复性

## 3 结论

本文采用巯基-烯点击反应制备了一种基于手性POC RCC3-R的新型HPLC CSP，该CSP在NP和RP色谱条件下均展现出较好的对映体分离性能，有18种外消旋体在NP洗脱模式下获得了拆分，16种外消旋体在RP洗脱模式下获得了拆分。与广泛应用的Chiralcel OD-H和Chiralpak AD-H商品柱相比，该RCC3-R色谱柱能够拆分一些在上述两种商品柱上无法拆分或者拆分效果不好的对映体，展现出了良好的手性识别互补性。本研究表明手性POCs是一种具有潜力的手性选择剂，该研究也对发展POCs类新型HPLC手性固定相具有重要的意义。
